# Safety and efficacy of *Melissa officinalis* extract containing rosmarinic acid in the prevention of Alzheimer’s disease progression

**DOI:** 10.1038/s41598-020-73729-2

**Published:** 2020-10-29

**Authors:** Moeko Noguchi-Shinohara, Kenjiro Ono, Tsuyoshi Hamaguchi, Toshitada Nagai, Shoko Kobayashi, Junji Komatsu, Miharu Samuraki-Yokohama, Kazuo Iwasa, Kunihiko Yokoyama, Hiroyuki Nakamura, Masahito Yamada

**Affiliations:** 1 Department of Neurology and Neurobiology of Aging, Kanazawa University Graduate School of Medical Sciences, Kanazawa University, 13-1 Takara-machi, Kanazawa, 920-8640 Japan; 2 Department of Preemptive Medicine for Dementia, Kanazawa University Graduate School of Medical Sciences, Kanazawa University, Kanazawa, Japan; 3 Division of Neurology, Department of Internal Medicine, Showa University School of Medicine, Hatano-dai, Shinagawa-ku, Tokyo, 142-8666 Japan; 4 Department of Food and Life-Science, Takasaki University of Health and Welfare, Gunma, Japan; 5 Research Center for Food Safety, Graduate School of Agricultural and Life Sciences, The University of Tokyo, Tokyo, Japan; 6 Department of Health and Medical Sciences, Ishikawa Prefectural Nursing University, Kahoku, Japan; 7 Department of Thyroidology, Public Central Hospital of Matto Ishikawa, Hakusan, Japan; 8 Department of Environmental and Preventive Medicine, Kanazawa University Graduate School of Medical Sciences, Kanazawa University, Kanazawa, Japan

**Keywords:** Diseases, Neurology

## Abstract

We conducted a randomized placebo-controlled double-blind 24-week trial using *Melissa officinalis* (*M. officinalis*) extract richly containing rosmarinic acid (RA) on patients with mild dementia due to Alzheimer’s disease (AD) with the aim to examine the safety and tolerability (primary endpoint) of RA (500 mg daily) and its clinical effects and disease-related biomarker changes (secondary endpoints). Patients (*n* = 23) diagnosed with mild dementia due to probable AD were randomized to either the placebo or *M. officinalis* extract group. No differences in vital signs or physical and neurologic examination results were detected between the *M. officinalis* and placebo groups. No serious adverse events occurred. There were no significant differences in cognitive measures; however, the mean Neuropsychiatric Inventory Questionnaire (NPI-Q) score improved by 0.5 points in the *M. officinalis* group and worsened by 0.7 points in the placebo group between the baseline and 24-week visit, indicating a significant difference (*P* = 0.012). No significant differences were apparent in disease-related biomarkers between the groups. *M. officinalis* extract containing 500 mg of RA taken daily was safe and well-tolerated by patients with mild dementia due to AD. Our results suggest that RA may help prevent the worsening of AD-related neuropsychiatric symptoms.

**Trial registration:** The registration number for this clinical trial is UMIN000007734 (16/04/2012).

## Introduction

Alzheimer’s disease (AD) is characterized by parenchymal and vascular amyloid deposits of amyloid β-protein (Aβ) and neurofibrillary tangles formed by the microtubule-associated protein tau^1^. Because the deposition of Aβ aggregates in the brain occurs approximately 25 years before the appearance of clinical symptoms, it has been proposed that treatments targeting Aβ aggregation, including formation of neurotoxic Aβ oligomers, should be implemented early in the disease process^2^. Dietary modifications and/or nutraceutical supplementation are appropriate methods for the prevention or treatment of AD.

Rosmarinic acid (RA) is an ester of caffeic acid and 3,4-dihydroxiphenyllactic acid. It has several interesting biological properties, including antioxidant, anti-inflammatory, antimutagenic, anti-bacterial, and antiviral properties^3^. We previously reported that RA inhibits the formation of Aβ fibrils, comprising Aβ_1-40_ and Aβ_1-42_, and destabilizes preformed Aβ fibrils in vitro in a dose-dependent manner^4^. RA also inhibits the oligomerization of both Aβ_1-40_ and Aβ_1-42_ in vitro^5^. Moreover, our long-term potentiation and depression assays using hippocampal slices indicated that RA decreases Aβ oligomer-induced synaptic toxicities^5^. Long-term potentiation and depression are considered important neurophysiological models of memory and learning and are used as experimental models of neuronal plasticity^6^. In intracerebroventricular Aβ_25-35_ injection mice model studies, intracerebroventricular injection of RA prevents memory impairment and Aβ-induced neurotoxicity by scavenging ONOO^–^^7^. We systematically investigated the effects of phenolic compounds on AD transgenic mice (Tg2576) model^8^. Mice were fed five phenolic compounds (curcumin, ferulic acid, myricetin, nordihydroguaiaretic acid, and RA) (1 g/kg/day) for 10 months from the age of 5 months^8^. Of the compounds tested, RA appeared to be the most attractive molecule for preventing AD, because it inhibited both the oligomerization and deposition of Aβ^8^. Moreover, we recently demonstrated that monoamine suppresses Aβ aggregation^9^, suggesting that an RA-triggered increase in monoamine secretion in the brain is beneficial for the treatment of AD.

RA is present in various herbs such as perilla (*Perilla frutescens L.*)^10^, rosemary (*Rosmarinus officinalis L.*)^11^, sage (*Salvia officinalis L.*)^12^, and lemon balm [*Melissa officinalis* (*M. officinalis*) *L.*]^13^. Extracts of *M. officinalis* are reported to have positive effects on cognitive performance in healthy participants^14,15^. In a 16-week, randomized, double-blind, placebo-controlled study, patients with mild to moderate AD treated with *M. officinalis* extract showed significantly higher cognitive function, with no side effects, compared with patients administered placebo^16^; however, the amount of RA contained in the *M. officinalis* extract was not analyzed, and no disease-related biomarkers were evaluated^16^. Previously, we prepared *M. officinalis* extract richly containing RA and showed that a single dose of *M. officinalis* extract containing 500 mg of RA is safe and tolerable in healthy individuals^17^.

Here, we performed a randomized double-blind placebo-controlled 24-week study of *M. officinalis* extract containing RA in patients with mild dementia due to AD, with an open-label extension to 48 weeks. The primary endpoint was the safety and tolerability of the *M. officinalis* extract, and the secondary endpoints the clinical efficacy of *M. officinalis* extract and changes in AD biomarkers.

## Results

### Primary outcome

A total of 23 patients were randomized to either the *M. officinalis* group (12 patients) or the placebo group (11 patients). Table 1 shows the demographic and clinical characteristics of patients included in the full-analysis set.

**Table 1:** Demographics and baseline clinical characteristics.

	*Melissa officinalis* extract group (n = 12)	Placebo group (n = 11)	P value
Age (years)	73.42 ± 5.00	72.45 ± 7.53	0.786
No. of women, n (%)	5 (41.6)	6 (54.5)	0.684
Years of education	11.92 ± 1.00	12.36 ± 2.20	1.000
APOE E4 carrier (%)	83.3	63.63	0.371
MMSE score	22.92 ± 1.83	23.55 ± 1.81	0.449
ADAS-cog score	25.92 ± 6.46	24.30 ± 4.37	0.456
DAD score	28.00 ± 8.83	26.27 ± 7.52	0.487
NPI-Q score	4.58 ± 3.63	4.82 ± 1.72	0.740
CDR score = 1 (%)	100	100	1.000
^11^C-PiB PET SUVR value	1.81 ± 0.21	1.81 ± 0.21	1.000
^18^F-FDG PET Z-score	2.07 ± 0.71	2.14 ± 0.82	0.833
Head MRI VSRAD value	3.87 ± 1.34	3.82 ± 1.21	0.976
CSF-Aβ_1-42_ concentration (pg/mL)	458.82 ± 140.80	447.78 ± 112.54	1.000
CSF-tau concentration (pg/mL)	828.80 ± 442.61	806.25 ± 516.82	0.829
CSF-ptau concentration (pg/mL)	95.09 ± 40.57	78.22 ± 32.06	0.295

Data represent mean ± standard deviation (SD) or percent values. Significant differences between groups were determined by analysis of variance (ANOVA) and Chi-square (χ^2^) test, where appropriate.

*Aβ* amyloid β-protein; *ADAS-cog* cognitive subscale of the Alzheimer’s Disease Assessment Scale; *APOE* apolipoprotein E; *CDR* clinical dementia rating; *CSF* cerebrospinal fluid; *DAD* Disability Assessment for Dementia; *FDG* fluorodeoxyglucose; *MMSE* Mini-Mental State Examination; *NPI-Q* Neuropsychiatric Inventory-Questionnaire; *PET* positron emission tomography; *ptau* phosphorylated tau 181p. *PiB* Pittsburgh compound-B; *SUVR* standardized uptake value ratio; tau, total tau protein; *VSRAD* Voxel-Based Specific Regional Analysis System for Alzheimer’s disease.

**Figure 1:**
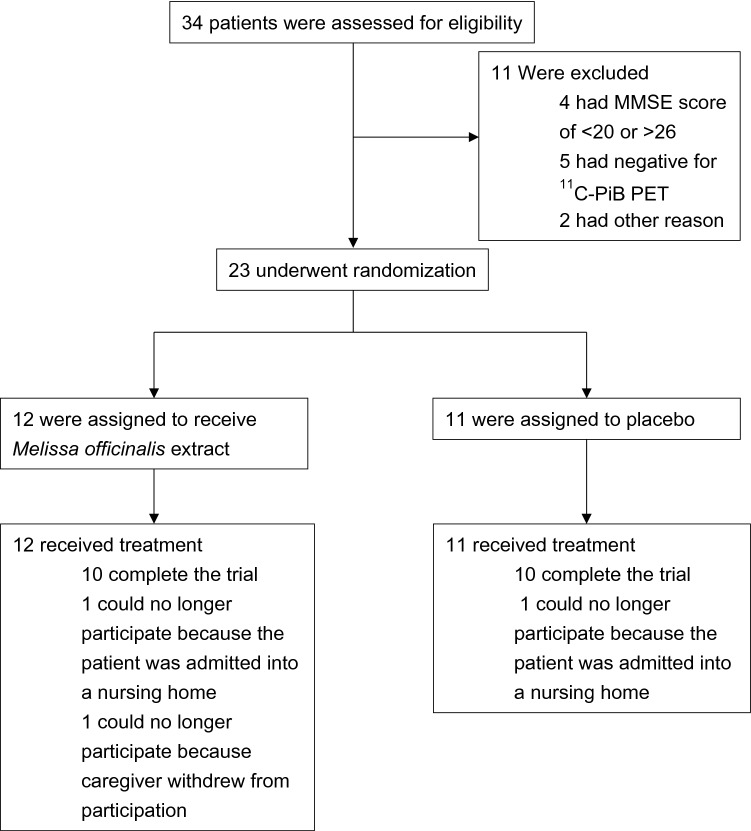
Randomization, trial-group assignment, and follow-up of the trial. Scores of the Mini-Mental State Examination (MMSE) range from 0 to 30, with lower scores indicating poorer cognitive performance. In the positron emission tomography (PET) study, the mean cortical standardized uptake value ratio (SUVR) was calculated using the ratio of the SUV between the whole cerebrum region of interest (ROI) and the reference cerebellum ROI. ^11^C-Pittsburgh compound-B (PiB) PET negative: mean cortical ^11^C-PiB SUVR values less than 1.45 indicated a negative result for Alzheimer’s disease (AD) pathology. *MMSE* Mini-Mental State Examination; *PET* positron emission tomography; *PiB* Pittsburgh compound-B; *ROI* region of interest; *SUVR* standardized uptake value ratio.

Three patients withdrew from the study between the baseline and 24-week follow-up visit; two patients (one from the *M. officinalis* group and one from the placebo group) withdrew because they had to be admitted into a nursing home, and another patient from the *M. officinalis* group withdrew because caregivers lost interest in the trial. A total of 20 patients (83.3–90.9% of the patients in each group) completed part 1 of the trial (Fig. 1 and Table 1), and all these patients entered part 2 (extension period).

No differences were detected in the vital signs and physical and neurological examinations between the *M. officinalis* and placebo groups. Overall, adverse events occurred in 41.6% of the patients in the *M. officinalis* group and 45.5% of the patients in the placebo group (*P* = 1.000). Regarding concomitant medications, no apparent adverse events were reported. None of these adverse events were serious or led to the discontinuation of the trial regimen (Table 2).

**Table 2:** Number of patients with adverse events.

Adverse events	*Melissa officinalis* extract group (*n* = 12)	Placebo group (*n* = 11)
Bruising	1	0
Headache	1	0
Hematuria	1	0
Upper respiratory infection	1	2
ALT elevation	0	1*
AST elevation	0	1*
Hyperkalemia	0	1
Hypokalemia	0	1
Rash maculopapular	0	2 (1*)
Weight loss	0	1

*ALT* alanine aminotransferase; *AST* aspartate aminotransferase.

*Adverse events were reported during the extension period.

Serum levels of liver function indicators [aspartate aminotransferase (AST), alanine aminotransferase (ALT), lactate dehydrogenase (LDH), and total bilirubin (T-bil)]and kidney function indicators [blood urea nitrogen (BUN) and creatinine (Cr)] and hematology findings showed no significant differences between the *M. officinalis* and placebo groups. Adherence to treatment of the *M. officinalis* and placebo groups were found to be 77.42% and 87.45%, respectively, in the full-analysis set.

### Secondary outcomes

The score changes from baseline to the 8-, 16-, and 24-week follow-up visits showed no significant differences in Mini-Mental State Examination (MMSE) cognitive subscale of the Alzheimer's Assessment Scale (ADAS-cog), Disability Assessment for Dementia scale (DAD) clinical dementia rating (CDR) between the treatment groups (Table 3).

**Table 3:** Mean and estimated mean changes in clinical assessments from the baseline to 24-week follow-up and the baseline to 48-week follow-up.

		Baseline	24 weeks	48 weeks	Mean change ΔBaseline-24 weeks	P value	Mean change ΔBaseline-48 weeks	P value
MMSE score	*M. officinalis* (*n* = 10) Placebo group (*n* = 10)	23.40 ± 1.58 23.70 ± 1.83	22.10 ± 2.73 22.40 ± 3.47	21.30 ± 3.86 23.30 ± 3.78	− 1.30 ± 2.36 − 1.30 ± 2.36	0.513	− 2.10 ± 3.73 − 0.40 ± 2.27	0.459
ADAS-cog score	*M. officinalis* (*n* = 10) Placebo group (*n* = 10)	23.90 ± 4.79 24.30 ± 4.37	23.60 ± 4.84 26.10 ± 6.17	26.30 ± 3.13 25.90 ± 8.63	− 0.30 ± 4.35 1.80 ± 3.88	0.239	2.40 ± 5.50 1.60 ± 5.32	0.657
DAD score	*M. officinalis* (*n* = 10) Placebo group (*n* = 10)	30.10 ± 7.31 27.90 ± 5.53	30.30 ± 5.74 27.40 ± 5.91	28.60 ± 5.73 25.20 ± 8.47	0.20 ± 1.99 − 0.50 ± 3.03	0.464	− 1.50 ± 4.50 − 2.70 ± 3.77	0.828
NPI-Q score	*M. officinalis* (*n* = 10) Placebo group (*n* = 10)	4.60 ± 3.84 4.80 ± 1.81	4.10 ± 3.51 5.50 ± 2.17	3.50 ± 3.03 6.10 ± 2.08	− 0.50 ± 1.43 0.70 ± 1.89	0.012	− 1.10 ± 2.54 1.30 ± 2.54	< 0.001
CDR score	*M. officinalis* (*n* = 10) Placebo group (*n* = 10)	1.0 (*n* = 10) 1.0 (*n* = 10)	1.0 (*n* = 10) 1.0 (*n* = 9) 2.0 (*n* = 1)	1.0 (*n* = 10) 1.0 (*n* = 9) 2.0 (*n* = 1)	–	–	–	–

*ADAS-cog* cognitive subscale of the Alzheimer’s Disease Assessment Scale; *CDR* clinical dementia rating; *DAD* Disability Assessment for Dementia; *M. officinalis*, *Melissa officinalis*; *MMSE* Mini-Mental State Examination; *NPI-Q* Neuropsychiatric Inventory-Questionnaire.

**Figure 2:**
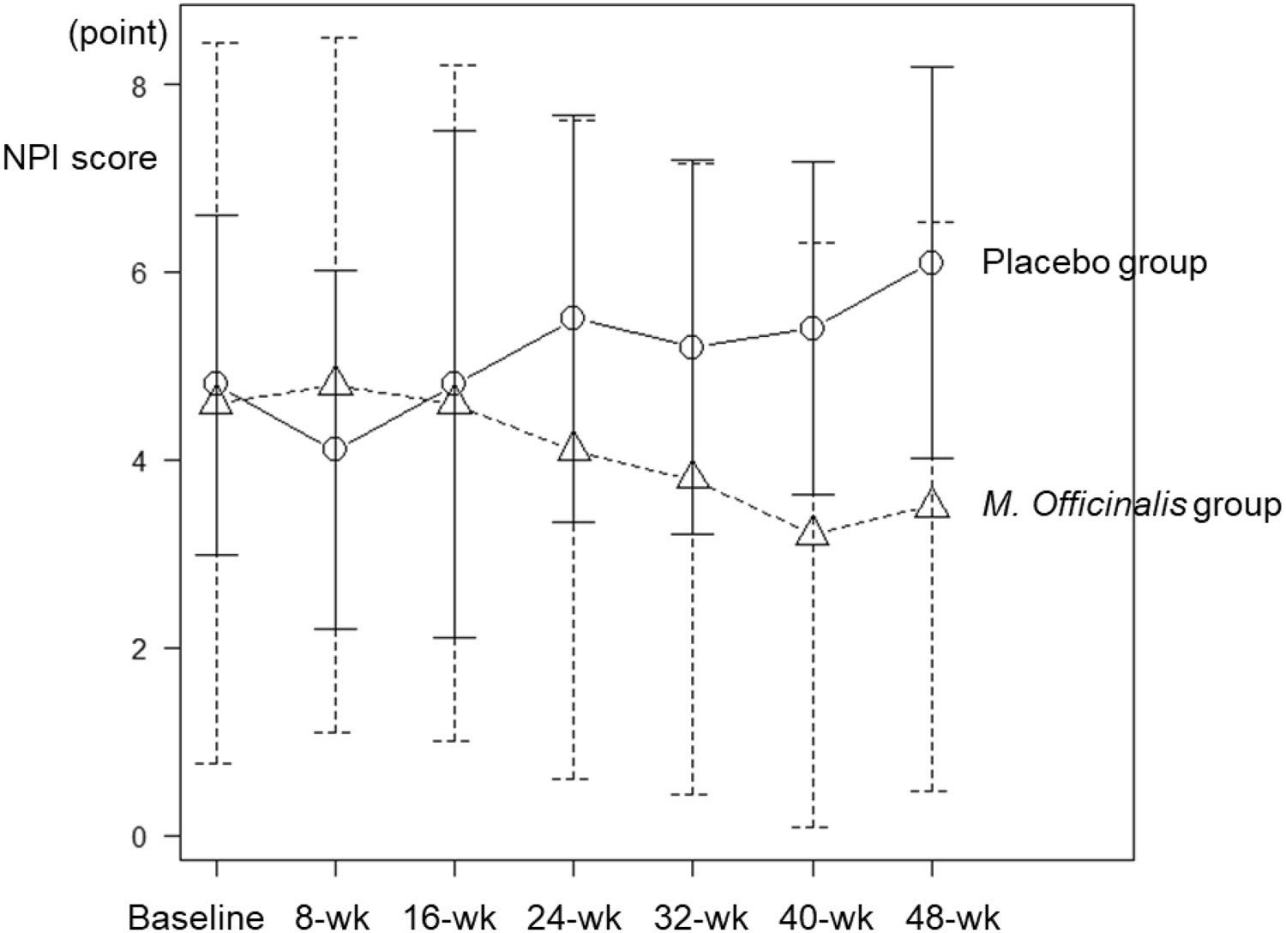
Means of Neuropsychiatric Inventory (NPI-Q) scores at each study time point.

Assessment of the neuropsychiatric effect of *M. officinalis* extract on the Neuropsychiatric Inventory-Questionnaire (NPI-Q) score from baseline to the 8-, 16-, and 24-week timepoints and baseline to the 8-, 16-, 24-, 32-, 40-, and 48-week timepoints using per protocol set revealed the time × treatment interaction effect as significant (*F* = 4.028, *P* = 0.012 and *F* = 5.766, *P* < 0.001, respectively; Fig. 2 and Table 3). Using full-analysis set, time × treatment interaction effects were significant for the NPI-Q score from baseline to 24-week follow-up and 48-week follow-up (*F* = 4.576, *P* = 0.006 and *F* = 5.984, *P* < 0.001), respectively. The data of each subject of the NPI-Q scores are summarized in the Supplemental Table.

The effect of time × treatment interaction on for NPI-Q scores was significant (*P* < 0.05). Open triangles represent the *M. officinalis* group, and open circles represent the placebo group.

The time variable was not significant (*F* = 0.484, *P* = 0.695). The mean NPI-Q scores improved by 0.5 points in the *M. officinalis* group and worsened by 0.7 points in the placebo group between baseline and the 24-week visit. On further analyzing each NPI-Q subscale, time × treatment variable of “Irritability/Lability” was significant (*F* = 4.539, *P* = 0.006); the mean scores of “Irritability/Lability” improved by 0.32 points in the *M. officinalis* group and worsened by 0.23 points in the placebo group between baseline and the 24-week visit. Regarding the extension period, in which all patients were assigned to the *M. officinalis* extract group, the mean NPI-Q scores worsened by 0.6 points in the placebo group between the 24-week visit and the 48-week visit.

Changes in disease-related biomarkers, including ^11^C-Pittsburgh compound-B (PiB) positoron emission tomography (PET) standardized uptake value ratio (SUVR), ^18^F-fluorodeoxyglucose (FDG) PET z-scores, MRI z-scores, and cerebrospinal fluid (CSF)-Aβ_1-42_, CSF-total tau protein (tau), and CSF-phosphorylated tau 181p (ptau) concentrations, showed no significant differences between the two treatment groups (Table 4).

**Table 4:** Mean and estimated mean changes in disease-related biomarker measures from the baseline to 24-week follow-up.

		Baseline	24 weeks	Mean change	*P* value
^11^C-PiB PET SUVR value	*M. officinalis* (*n* = 10) Placebo (*n* = 9)	1.81 ± 0.22 1.80 ± 0.19	1.76 ± 0.26 1.79 ± 0.35	− 0.05 ± 0.10 − 0.02 ± 0.30	0.838
^18^F-FDG PET Z score	*M. officinalis* (*n* = 10) Placebo (*n* = 10)	1.91 ± 0.65 2.00 ± 0.70	2.01 ± 0.75 2.26 ± 0.97	0.10 ± 0.19 0.26 ± 0.51	0.352
Head MRI VSRAD value	*M. officinalis* (*n* = 10) Placebo (*n* = 10)	3.59 ± 1.29 4.02 ± 1.06	3.70 ± 1.53 4.35 ± 1.22	0.12 ± 0.41 0.33 ± 0.47	0.286
CSF-Aβ_1-42_ concentration	*M. officinalis* (*n* = 7) Placebo (*n* = 6)	507.86 ± 142.09 492.17 ± 101.64	490.57 ± 52.73 435.50 ± 60.98	− 17.29 ± 124.75 − 56.67 ± 75.39	0.559
CSF-tau concentration	*M. officinalis* (*n* = 6) Placebo (*n* = 6)	922.17 ± 460.45 924.50 ± 552.37	959.67 ± 527.59 882.67 ± 487.78	37.50 ± 141.19 − 41.83 ± 180.65	0.367
CSF-ptau concentration	*M. officinalis* (*n* = 7) Placebo (*n* = 6)	106.14 ± 41.44 91.17 ± 31.92	114.57 ± 41.60 95.33 ± 29.81	8.43 ± 17.19 4.17 ± 6.85	0.558

*Aβ* amyloid β-protein; *CSF* cerebrospinal fluid; *FDG* fluorodeoxyglucose; *M. officinalis*, *Melissa officinalis*; *PET* positron emission tomography; *PiB* Pittsburgh compound-B; *ptau* phosphorylated tau 181p; *SUVR* standardized uptake value ratio; *tau* total tau protein; *VSRAD* Voxel-Based Specific Regional Analysis System for Alzheimer’s disease.

### Exploratory examination

**Figure 3:**
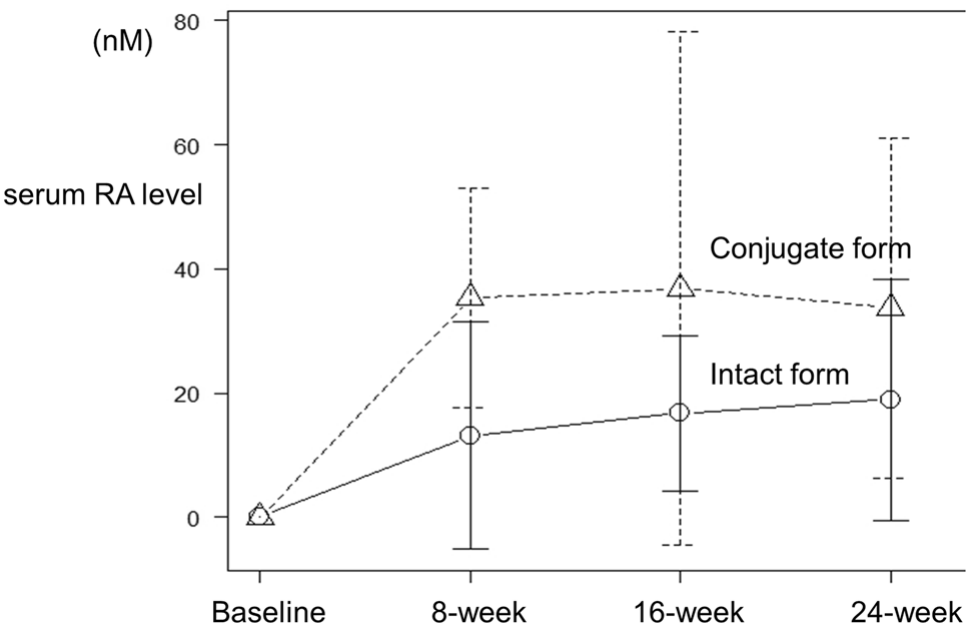
Mean serum RA levels at each study time point in the *M. officinalis* group. Open triangles represent the conjugated form of rosmarinic acid (RA), and open circles represent the intact form of RA.

The mean concentrations of total RA (intact and conjugated forms) in serum samples of the *M. officinalis* group at the 8-, 16-, and 24-week visits were 52.60 ± 41.38, 48.49 ± 27.83, and 53.58 ± 46.55 nmol/L, respectively (Fig. 3). Conjugated form of RA reached peaked at 16 weeks and slightly decreased at 24 weeks, while intact form of RA gradually increased up to 24 weeks.

RA was not detectable in the serum at the baseline (prior to treatment with *M. officinalis* extract) in the intervention group or at any time point during the trial period in the placebo group, except in the serum collected from one subject in the placebo group; surprisingly, in this subject, total RA in the serum at the baseline, 8-, 16-, and 24-week visits was 30.63, 30.06, 13.20, and 47.22 nmol/L, respectively. Additionally, in one *M. officinalis* group patient, RA in the serum could be detected only at the 16-week visit (32.58 nmol/L) but not at the 8- and 24-week visits, as the concentrations were below the level of detection; the study partner of this patient reported adherence as 88.00%. RA levels were undetectable (< 0.28 × 10^−2^ nmol/L) in the CSF of patients in both the placebo and *M. officinalis* groups at the baseline and the 24-week follow-up.

## Discussion

In the 24-week double-blind placebo-controlled study, with a 24-week extension, we examined the safety and tolerability of *M. officinalis* extract containing RA (500 mg daily dose) in patients with mild dementia due to AD as a primary endpoint and demonstrated that supplementation with this dose was tolerable for 48 weeks and did not raise any safety concerns that manifested clinically or through routine blood tests.

We examined the influence of *M. officinalis* extract containing RA on clinical assessments and biomarkers as secondary endpoints. No differences were detected between the treatment and placebo groups in terms of cognitive performance or daily function between the baseline and 24-week follow-up visits; however, a significant time × treatment group interaction was observed for the NPI-Q score and its subscale “Irritability/Lability”, suggesting benefits of the *M. officinalis* extract in the management of irritability or lability in patients with AD. Consistent with our results, *M. officinalis* extract and aromatherapy with the essential oil of *M. officinalis* have been shown to have a positive effect on agitation in patients with mild to moderate AD and severe dementia, respectively^16,18^. In addition, *M. officinalis* essential oil was reported to decrease NPI scores in patients without dementia, implying that it reduces agitation^19^. *M. officinalis* has both nicotinic and muscarinic acetylcholine receptor binding activity; however, the specific action of *M. officinalis* on acetylcholine receptors remains unknown^20^, and whether it shows efficacy in the treatment of patients with AD with respect to not only their behavioral and psychological symptoms of dementia, such as irritability, but also their cognition is unclear^20^. Further, we were unable to reveal biological evidence of efficacy against AD pathophysiology about changes in amyloid or tau markers in this trial with about 10 patients in each group. Overall, we were able to provide a little evidence for the clinical efficacy of *M. officinalis* extract as the secondary outcomes of this study.

This is the first randomized double-blind placebo-controlled study conducted to evaluate RA levels in the serum and in CSF in humans. Interestingly, two participants showed unexpected serum RA levels in the placebo and *M. officinalis* groups. One patient in the placebo group showed sufficient serum RA concentration. Because some Japanese individuals consume *Perilla frutescens*^10^, which is rich in RA, our data indicate that this patient might have been eating *Perilla frutescens*. Further trials should exclude subjects who consume any diets rich in RA during the trial. On the contrary, one subject in the *M. officinalis* group had considerably low or undetectable levels of serum RA; it is possible that this participant may have had poor medication compliance, in contrast to that reported of the patient’s study partner. Our previous study suggests that RA is metabolized and degraded by gut microbiota^21^; therefore, the pharmacokinetics of RA may be affected by the individual states of microbiota. Blood and CSF samplings were performed 4 or 5 h after the intake of *M. officinalis* extract containing RA (500 mg daily dose). In the *M. officinalis* group, at the 24-week visit, the serum level of RA and its metabolites was 53.58 ± 46.55 nmol/L, but the CSF level of RA was below detectable levels (< 0.28 × 10^−2^ nmol/L). Time taken to reach the maximum serum concentration of RA in fed state was reported to be 3 h after the intake of *M. officinalis* extract containing RA^17^; higher levels of RA in serum and CSF might have been observed if serum and CSF were collected at an earlier timing. Pharmacokinetic study revealed that conjugated form of RA in serum reached peak at 16 weeks and slightly decreased at 24 weeks, while intact form of RA in serum gradually increased up to 24 weeks. This result might suggest that conjugation ability reached maximum. RA was reported to act as a weak or moderate inhibitor of drug-metabolizing enzymes, such as cytochrome P450 monooxygenase (CYP) uridine and diphosphate glucuronosyltransferase^22^. In the present study, we did not observe any apparent interactions between *M. officinalis* extract and concomitant medications. However, as acetylcholinesterase inhibitors are metabolized by CYP3A4 and CYP2D6^23^, we must pay attention to interactions between RA and concomitant medications especially when patients are treated with anti-AD drugs. Further investigations are necessary to clarify interactions between RA and drugs.

We consider that *M. officinalis* extract containing RA exerted its effects in the brain to prevent the worsening of neuropsychiatric symptoms, although concentrations of RA were considerably low in the CSF. Studies using isotope labeled RA is presumed to be useful to clarify the absorption and distribution of RA in blood, CSF and brain following oral administration. However, as far as we investigated, there have been no studies which show that isotope-labeled RA appears in the central nervous system following oral administration. A previous study with rats demonstrated that intact RA is present in the plasma and brain at mean concentrations of 1440 and 24.1 μM, respectively (plasma:brain = 60:1), at 30 min after the intraperitoneal administration of *Plectranthus barbatus* extract (containing 250 μM RA kg^−1^)^24^. It was reported that the ratio of the mean of CSF concentration (C_CSF_) to that of brain interstitial fluid concentration (C_ISF_) (C_CSF_/C_ISF_) for propranolol was 0.49 in nonhuman primates, indicating that C_ISF_ was greater than C_CSF_^25^. Additionally, concentration of drug in the brain to C_CSF_ ratios was reported to be compound-dependent in rats^26^. Although we did not determine either the C_ISF_ of RA or the RA concentrations in the brain in this study, it is possible that the C_ISF_ of RA and the RA concentrations of brain might be higher than that of C_CSF_.

RA might act to suppress AD development even at considerably low concentration in the brain. In our in vivo study of an AD mouse model, RA inhibited Aβ oligomerization and deposition^8^. In addition, we recently reported RA-triggered monoamine increase in the brain, and inhibition of Aβ aggregation by monoamines in mice fed with RA^9^; however, RA concentrations in the brain of these mice^8,9^ were undetectable. Another possibility is that RA might act on peripheral targets such as gastrointestinal tract, which might contribute to prevention of the worsening of neuropsychiatric symptoms^27^.

The small sample size in this study warrants caution in the interpretation of results and limits their generalization. RA has been reported to decrease Aβ oligomer-induced synaptic toxicities^5^. Because pathogenic Aβ oligomers appear early in the disease^28^, treatment of AD using RA should be employed earlier in the disease process; for example, at the preclinical stage. A community-based intervention trial using *M. officinalis* extract containing RA aimed at the prevention of dementia is currently ongoing^29^.

This randomized double-blind placebo-controlled study on patients with mild AD dementia demonstrated that *M. officinalis* extract containing RA (500 mg daily dose) was safe and well-tolerated and that RA may provide a little help to control the worsening of neuropsychiatric symptoms in mild AD dementia.

## Materials and methods

### Patient population

Patients with mild dementia due to AD were enrolled at the Kanazawa University Hospital. The inclusion criteria were as follows: (i) diagnosis of probable AD dementia with high evidence of the pathophysiological process, according to the criteria of the National Institute on Aging-Alzheimer’s Association Workgroup (NIA-AA)^30^; (ii) age greater than 59 years; (iii) MMSE scores^31^ between 20 and 26; (iv) CDR score^32^ of 0.5 or 1 (the CDR score ranges from 0 to 3, with higher scores indicating worsening dementia); (v) availability of a study partner to monitor the administration of medication; (vi) stable dosage of acetylcholinesterase inhibitors and memantine for 60 days before screening. The exclusion criteria were as follows: (i) a significant systemic illness such as liver, renal, or heart dysfunction, (ii) a history of cancer, (iii) allergies to polyphenols or any drug or food ingredient, and (iv) subjects who consume any supplements containing RA. Additionally, magnetic resonance imaging (MRI), ^11^C-PiB PET, and ^18^F-FDG PET were performed to exclude patients with alternative causes of dementia. Furthermore, the number of apolipoprotein E (APOE) E4 carriers were evaluated in the placebo and *M. officinalis* groups. The APOE phenotype was determined using isoelectric electrophoresis, as described by Kamboh et al.^33^.

This study was conducted according to the guidelines of the Declaration of Helsinki, and all procedures involving human participants were approved by the Kanazawa University Medical Ethics Review Board (approval number 932). The study was registered at the University Hospital Medical Information Network Clinical Trials Registry (UMIN-CTR: UMIN000007734) (16/04/2012). Written informed consent was obtained from all patients or their legal representatives. An independent data monitoring committee (IDMC) monitored the progress of the trial.

### Intervention

**Figure 4:**
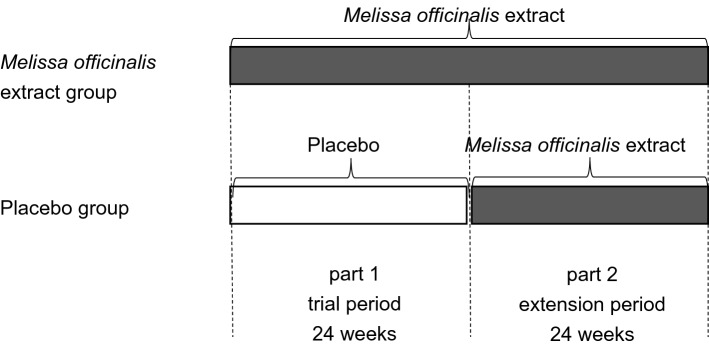
Trial time course. Part 1 of the trial was designed as a randomized double-blind placebo-controlled parallel-group 24-week trial. Patients were randomly allocated in a 1:1 ratio to either the *Melissa officinalis* group [receiving *M. officinalis* extract containing 500 mg of rosmarinic acid (RA) daily] or the placebo group. In part 2 (duration of 24 weeks), all patients were assigned to the *M. officinalis* extract group.

This study consisted of a randomized double-blind placebo-controlled parallel-group 24-week trial period (part 1), followed by an extension period of 24 weeks (part 2). In part 2, all patients were allocated to the *M. officinalis* group. Figure 4 illustrates the time course of the trial.

Study visits were set up at screening, baseline, and 8-, 16-, 24-, 32-, 40-, and 48-week follow-ups. Randomization was stratified according to age and gender. All patients were randomly allocated in a 1:1 ratio to receive the intervention (*M. officinalis* extract) or placebo. Block randomization was used to maintain a balance of three age-strata (60–69, 70–79, and > 80 years of age) and gender.

### Primary outcome measures

The primary outcome was the safety and tolerability of *M. officinalis* extract containing RA on ingestion. Safety assessments included the vital sign evaluation, physical and neurological examinations, adverse event evaluation, urinalysis, and routine laboratory tests, including hematology (white blood cell count, red blood cell count, hemoglobin level, hematocrit, and platelet count) and blood biochemistry analysis (levels of AST, ALT, LDH, T-bil, BUN, and Cr), electrocardiography, and chest X-rays. Blood samples were collected before lunch. Most of the patients ingested the placebo or *M. officinalis* extract capsules 4 or 5 h before blood sample collection. CSF was collected at the same time as blood samples. All safety information was reviewed by the IDMC at quarterly intervals. Unused *M. officinalis* extract or placebo capsules were stored by the patients, brought back to the investigating physician during each follow-up consultation, and counted to measure adherence.

### Secondary outcome measures

The secondary outcomes included changes in clinical assessments and biomarkers. An array of cognitive and neuropsychiatric measures was administered at baseline and during the 8-, 16-, and 24-week follow-up assessments. The measures included an ADAS-cog (score range = 0–70, with higher scores indicating worsening dementia)^34^, MMSE, assessment of dementia according to the CDR, assessment of daily function according to the DAD (score range = 0–40, with lower scores indicating worsening dementia)^35^, and NPI-Q (score range = 0–36, with higher scores indicating more severe symptoms)^36^. Biomarkers included ^11^C-PiB PET, ^18^F-FDG PET, volumetric MRI, and CSF biochemical markers [Aβ_1-42_, tau, and ptau]. The change from the baseline to the 24-week follow-up visit was assessed based on the ^11^C-PiB SUVR^37,38^, ^18^F-FDG PET z-scores (the degree of regional metabolic reduction)^39,40^, MRI z-scores (the degree of atrophy in the entorhinal cortex)^41^, and CSF markers^42,43^. Details of methods for PET, MRI and CSF marker studies are summarized in Additional Method 1.

### Exploratory examination

Concentrations of intact and conjugated RA were assessed in the serum at baseline and during the 8-, 16-, and 24-week follow-up visits, and in CSF at baseline and the 24-week follow-up. RA levels in the serum were measured using high-performance liquid chromatography (HPLC), as described previously^44,45^, with the lower limit of detection as 10 nmol/L. RA levels in the CSF were measured using liquid chromatography coupled with electrospray ionization tandem mass spectrometry (LC–ESI–MS/MS). The limit of detection for intact and conjugated RA in the CSF samples was 0.28 × 10^−2^ nmol/L. Details of quantification of RA levels in the serum and CSF are summarized in Additional Method 2.

### Statistical analyses

The sample size was determined based on the primary objective, i.e., to assess the safety and tolerability of *M. officinalis* extract containing RA.

Measurements used to determine the safety of *M. officinalis* extract containing RA were analyzed using all the available data from all available subjects (full-analysis set). Analysis of the efficacy of *M. officinalis* extract containing RA was performed using data collected from patients who completed the whole study because of the lack of follow-up biomarker data for subjects who discontinued the project (per protocol set) in addition to full-analysis set. Generalized linear mixed effects repeated measures analysis was used to analyze the effect of treatment on changes in all measures from baseline to the 8-, 16-, 24-, 32-, 40-, and 48-week follow-up visits. Data were reported as mean ± standard deviation, unless specified otherwise. A *P* value of < 0.05 was considered statistically significant. All statistical analyses were performed using the SPSS software (version 23; SPSS Inc., Chicago, IL, USA).

## Supplementary information

Supplementary Information.

## Data Availability

The datasets used and analyzed during the current study are available from the corresponding author on reasonable request.

## Code availability

The full trial protocol can be accessed in https://upload.umin.ac.jp/cgi-open-bin/ctr/ctr_view.cgi?recptno=R000009110 (English and Japanese).
