# Supplementary material for: Safety and efficacy of Melissa officinalis extract containing rosmarinic acid in the prevention of Alzheimer’s disease progression
Source: Sci Rep. 2020 Oct 29;10:18627. doi: 10.1038/s41598-020-73729-2 (PMC7596544; doi:10.1038/s41598-020-73729-2)
Supplement: Supplementary file 1 — Supplementary Information. [file 41598_2020_73729_MOESM1_ESM.pdf]

Safety and efficacy of *Melissa officinalis* extract containing rosmarinic acid in the prevention of Alzheimer's disease progression

Moeko Noguchi-Shinohara, MD, PhD; Kenjiro Ono, MD, PhD; Tsuyoshi Hamaguchi, MD, PhD; Toshitada Nagai PhD; Shoko Kobayashi, PhD; Junji Komatsu, MD, PhD; Miharu Samuraki-Yokohama, MD, PhD, Kazuo Iwasa, MD, PhD; Kunihiko Yokoyama MD, PhD; Hiroyuki Nakamura, MD, PhD; Masahito Yamada, MD, PhD

Supplementary method 1

PET methods

The brain amyloid load was assessed by PET using ^{11}C-PiB. ^{11}C-PiB PET imaging was performed with a full-ring PET scanner (Advance, GE Healthcare, Milwaukee, WI). After positioning the subject in a supine position in the PET gantry, 555 MBq of ^{11}C-PiB was intravenously injected and 5 min data acquisition was repeated four times starting from 50 min after injection. The image data were acquired in 128 × 128 matrices with the pixel size of 2.11 mm and a slice thickness was 4.25 mm. Both attenuation and scatter corrections were employed during image reconstruction. The PET images were then smoothed using a 12-mm full-width at half-maximum (FWHM) isotropic Gaussian kernel. After completion of the emission scan, a transmission scan was performed for 10 min using ^{68}Ge/^{68}Ga pin sources for correction of attenuation. PET were co-registered to each patient's MR image and spatially normalized using parameter files that normalize the MR images to a default template on SPM8. To calculate the SUVR, a circular region of interest (ROI) was used as the reference cerebellar cortical ROI and the whole cerebrum ROIs were generated using WFU Pickatlas (Department of Radiology of Wake Forest University School of Medicine, Winston-Salem, NC, USA; fmri.wfubmc.edu) (Tzourio-Mazoyer N, et al. NeuroImage. 2002;15:273-89). The mean cortical SUVR was calculated by determining the SUVR between the whole cerebrum ROI and the reference cerebellar ROI. A previous study suggested that mean cortical ^{11}C-PiB SUVR values less than 1.45 indicated a negative for AD pathology (Mormino EC, et al. Brain. 2008;132:1310-23). In this study, we considered ^{11}C-PiB SUVR values greater than 1.50 to be positive for AD pathology.

For ^{18}F-FDG PET, 4.4 MBq/kg of ^{18}F-FDG were injected intravenously and subjects were kept at rest for an additional 45 min; then, PET imaging was then initiated for 30 min in 3-D mode. The PET scanner used and data acquisition method were exactly the same as ^{11}C-PiB

PET imaging. All ^{18}F-FDG PET images were spatially normalized to a standardized stereotactic space based on the MNI Atlas used as a default template for SPM8. Each of the processed ^{18}F-FDG PET images was compared with the mean and standard deviation (SD) of the PET image made from the normal database, which was generated in the same manner as our previous study (Matsunari I, et al. J Nucl Med. 2007;48:1961-70); this was through voxel-by-voxel z-score analysis using a software program developed by Matsuda (Matsuda H. J Nucl Med. 2007;48:1289-300). The z-score equals [(control mean) - (individual value)] /(control SD) as previously reported by Minoshima et al (Minoshima S, et al. J Nucl Med. 1995;36:1238-48).

MRI methods

MRI studies were performed using a 1.5-T system (Sigma Horizon; GE Healthcare). Three-dimensional volumetric acquisition of a T1-weighted gradient echo sequence at 10.74/4.848 (TR/TE) produced a gapless series of contiguous, thin sagittal sections with the following parameters: flip angle, 25°; acquisition matrix, 256 × 256; field of view, 25.0 cm; section thickness, 1.4 mm (Samuraki M, et al. Eur J Nucl Med Mol Imaging 2007; 34: 1658-1669). Voxel-Based Specific Regional Analysis System for Alzheimer's Disease (VSRAD) analysis was performed using VSRAD plus software (VSRAD plus, Eisai Co., Ltd. Japan). The z-score was calculated as previously reported (Hirata Y, et al. Neuroscience lett. 2005;382:269-74).

Methods for measurement of CSF markers

Samples of CSF were collected at the baseline visit and at the 24-week visit. All samples were centrifuged and frozen at −80°C. Sandwich enzyme-linked immunosorbent assays (ELISA) were used to determine CSF-Aβ_{1-42} (Innotest β-amyloid (1–42); Fujirebio,

Belgium), CSF-tau (Innotest hTAU-Ag; Fujirebio), and CSF-ptau (Innotest Phospho-tau (181p); Fujirebio) as previously described (Maddalena A. et al., Arch Neurol 2003;60:1202-1206, Hulstaert F. et al., Neurology 1999;52:1555-1562). The CSF-ptau was measured for an epitope of the phosphorylated 181st amino acid of tau protein (Maddalena A. et al., Arch Neurol 2003).

Supplementary method 2

Quantification of RA levels of serum

To measure intact RA, a 50 μL of serum were added to 50 μL of 0.1 mol/L sodium acetate buffer (pH 5.0) and 200 μL of 0.83 mol/L acetic acid in methanol. The mixture was vortexed, sonicated, and centrifuged (at 8500 × g for 5 min at 4°C) as previously described (Noguchi-Shinohara M, et al. PLoS ONE. 2015; 10: e0126422). The supernatant was injected onto a high-performance liquid chromatography (HPLC)-electrochemical detector C18 column (ODS150, MC Medical, Inc., Tokyo, Japan). HPLC was carried out with coulometric electrochemical detection (ESA Inc., Boston, USA). The quantitative determination of RA was performed using an external standard method (Konishi Y, et al. J Agric Food Chem. 2005;53:4740-6), which verified that the detector response was linear for an RA concentration. Mobile phase A (solvent A) was 50 mmol/L sodium acetate containing 5% methanol (pH 3.0 adjusted with phosphoric acid), while the mobile phase B (solvent B) was 50 mmol/L sodium acetate containing 40% acetonitrile and 20% methanol (pH 3.5 adjusted with phosphoric acid) (Noguchi-Shinohara M, et al. PLoS ONE. 2015; 10: e0126422). We used the following elution profile (0.6 mL/min): 0–28.5 min, linear gradient from 85% solvent A/15% solvent B to 20% solvent A/80% solvent B; 28.5–31 min, isocratic elution with 0% solvent A/100% solvent B; and 31–35 min, isocratic elution with 85% solvent A/15% solvent B. The eight electrode detector potentials were increased from 0 to 700 mV in increments of 100mV (Noguchi-Shinohara M, et al. PLoS ONE. 2015; 10: e0126422). A previous report determined that the majority of ingested RA is present as conjugated forms in the blood (Baba S, et al. Eur J Nutr. 2005;44:1-9). To measure sulfate and glucuronide conjugates of RA, serum (50 μL) was mixed with 50 μL of glucuronidase type H-5 (Sigma-Aldrich. Inc. St. Louis. MO. USA) solution in 0.1 mol/L acetate buffer (pH 5.0) containing 1.3 units of sulfatase and ~200 units of β-glucuronidase activity (Noguchi-

Shinohara M, et al. PLoS ONE. 2015; 10: e0126422). The mixture was incubated at 37°C for 45 min. The supernatant was injected onto a HPLC and analyzed using the same procedure as for the intact RA. The difference in RA content with and without glucuronidase treatment was assumed to be the amount of conjugated RA (Noguchi-Shinohara M, et al. PLoS ONE. 2015; 10: e0126422).

Quantification of RA levels of CSF

To measure intact RA, a 100 μL aliquot each CSF sample was spiked with 200 μL of 0.83 mol acetic acetate/100% methanol. The mixture was vortex-mixed for 1 min. After centrifugation at 8,500 × g for 5 min, 2 μL of supernatant was assessed by liquid chromatography coupled with electrospray inonization tandem massspectrometry (LC-ESI-MS/MS). Regarding LC-ESI-MS/MS, a 3200Q TRAP tandem mass spectrometer (AB Sciex, Framingham, MA, USA) equipped with a CAPCELL CORE C18 column (2.7 µm, 50 mm × 2.1 mm id, Osaka Sods Co., Ltd.). The mobile phase for LC consisted of acetonitrile containing 0.1% acetic acid (solvent A) and water containing 0.1% acetic acid (solvent B) in a linear gradient at a flow rate of 0.2 ml/min. The gradient program was as follows: 0 to 3 min, 20 to 50% A; 3 to 6 min, 90% A; 6 to 10 min, 20% A. The instrument was operated in negative ionization mode with automatically optimized parameters. Multiple reaction monitoring was employed for data acquisition. The presence of RA was assessed by selecting *m/z* 359.0 as the precursor ion and *m/z* 160.1 or *m/z* 197.0 as the fragmented ions. To measure sulfate and glucuronide conjugates of RA, CSF was also treated with glucuronidase type H-5 (Sigma-Aldrich) using the same procedure as for the serum and then analyzed using LC-ESI-MS/MS.

Supplemental table. The Neuropsychiatric Inventory-Questionnaire scores of each subject

Group	Subject	PPS	Baseline	8 weeks	16 weeks	24 weeks	32 weeks	40 weeks	48 weeks
Melissa officinalis extract	1	PPS	2	1	1	1	1	1	1
	2	PPS	11	11	11	10	9	9	9
	3	PPS	7	7	6	6	5	3	3
	4		7	9	ND	ND	ND	ND	ND
	5	PPS	10	10	10	10	10	8	8
	6	PPS	2	3	3	3	3	3	3
	7		2	ND	ND	ND	ND	ND	ND
	8	PPS	6	5	4	4	4	4	4
	9	PPS	2	2	2	2	1	1	3
	10	PPS	1	3	2	3	3	3	0
	11	PPS	0	0	1	0	0	0	0
	12	PPS	5	6	6	2	2	0	4
Placebo	1	PPS	7	4	6	7	5	5	6
	2		5	4	8	ND	ND	ND	ND
	3	PPS	5	4	5	5	5	5	7
	4	PPS	5	3	2	2	2	1	1
	5	PPS	1	0	0	4	3	5	6
	6	PPS	3	3	2	2	3	5	5
	7	PPS	5	5	6	6	6	6	7
	8	PPS	6	6	9	8	8	7	7
	9	PPS	7	7	7	7	7	7	7
	10	PPS	4	4	6	7	6	6	6
	11	PPS	5	5	5	7	7	7	9

PPS, per protocol set. ND, no data.

Supplementary figure

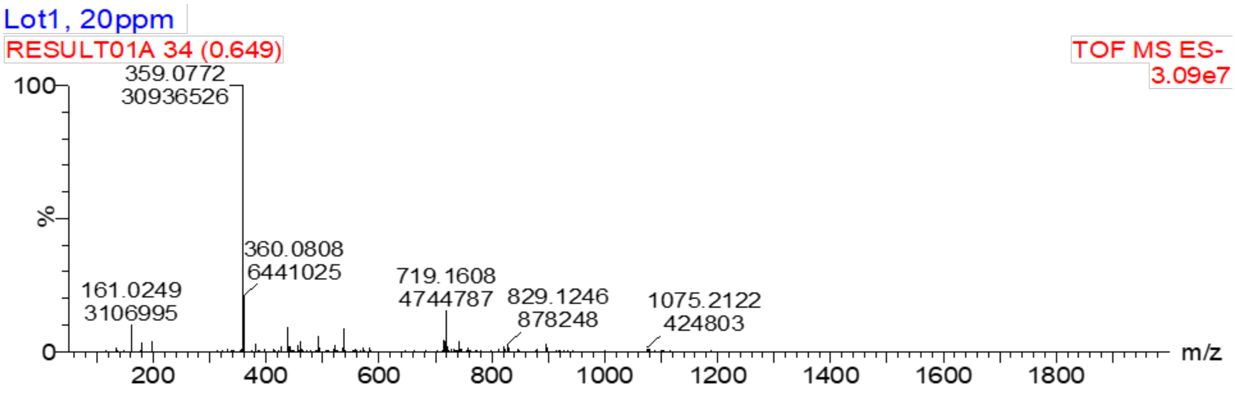

Figure legend

ESI-MS spectrum was obtained in negative ion mode on a TOF mass spectrometer; Xevo G2 Tof MS (Waters). The signal at m/z 359.0772 was assumed to be deprotonated rosmarinic acid ion.
